# Enhanced DBR mirror design via D3QN: A reinforcement learning approach

**DOI:** 10.1371/journal.pone.0307211

**Published:** 2024-08-22

**Authors:** Seungjun Yu, Haneol Lee, Changyoung Ju, Haewook Han

**Affiliations:** Department of Electrical Engineering, Pohang University of Science and Technology (POSTECH), Pohang, Republic of Korea; Rutgers University Newark, UNITED STATES OF AMERICA

## Abstract

Modern optical systems are important components of contemporary electronics and communication technologies, and the design of new systems has led to many innovative breakthroughs. This paper introduces a novel application based on deep reinforcement learning, D3QN, which is a combination of the Dueling Architecture and Double Q-Network methods, to design distributed Bragg reflectors (DBRs). Traditional design methods are based on time-consuming iterative simulations, whereas D3QN is designed to optimize the multilayer structure of DBRs. This approach enabled the reflectance performance and compactness of the DBRs to be improved. The reflectance of the DBRs designed using D3QN is 20.5% higher compared to designs derived from the transfer matrix method (TMM), and these DBRs are 61.2% smaller in terms of their size. These advancements suggest that deep reinforcement learning, specifically the D3QN methodology, is a promising new method for optical design and is more efficient than traditional techniques. Future research possibilities include expansion to 2D and 3D design structures, where increased design complexities could likely be addressed using D3QN or similar innovative solutions.

## Introduction

The design of efficient and compact optical systems has been considered critical for the development of modern electronic and telecommunication components [1–4], and is expected to play an important role in future technology advancement and innovation. In particular, the field of nano-optics has developed considerably in recent decades. These developments have centered on the fabrication of 2D and 3D metasurfaces [5, 6] or metamaterials [7] with outstanding characteristics that are not found in nature, and were the result of the combination of fundamental characteristics to produce optical components that are capable of controlling light very precisely. These achievements became possible because of the advancement in nanoscale fabrication techniques [8–11] and by fine-tuning periodically structured materials.

In the past ten years, optimized algorithms and numerical methods to design optical devices by directly calculating the interaction between light and materials have been proposed as new approaches to device design [12–17]. Although these methods do not require their users to have intuitive capabilities, the methods proved to be effective tools to develop optimized solutions to respond to specific requirements. More recently, studies aiming to introduce machine learning to design optical components have attracted more attention, and included the design of optical analog accelerators [18–25] and physical emulators [26]. Reverse designing, in which optical structures are proposed by studying the given optical responses, is possible with artificial intelligence models [27–35]. These methods, which include heuristic swing [36], genetic algorithms [37, 38], and neighbor-based topology optimization [39, 40], have improved the performance of optical components by increasing their efficiency and accuracy compared to other nano-optical devices [41–43].

Traditional design methods enabled nanostructures to be optimized based on time-consuming iterative simulations [44]. These optimization methods are sensitive to the initial values, the number of calculations for each case, and may not converge. Attempts to overcome these problems have recently led to the proposal of design methods based on deep learning [44]. Nano-optical structures, which have high optical performance in the nano-region, have been designed using neural networks based on these deep-learning methods [45–48]. Although optical structures with the required responses could be designed and proposed using deep learning, methods based on this technique require a large amount of learning data and are computationally intensive [49]. Generally, a large amount of optical response data is generated by using simulation methods such as rigorous coupled-wave analysis(RCWA), finite element method (FEM), and finite-difference time-domain method(FDTD), all of which are time consuming and computationally costly [32].

Recently, gradient-based optimization approaches were used to design optical components and were shown to exhibit excellent space light distributions dependent on the wavelengths [1, 50, 51]. However, the proposed 2D model has limitations in that it is not able to fully reflect the actual physics to provide practical device designs [1].

Herein, we propose an optical component design technique similar in concept to the above. However, the proposed technique is based on a contrasting theoretical realization motivated by the gradient method according to the concept of reinforcement learning [52, 53]. Toward this end, the variation problems and the need for a large amount of learning data, both of which were prerequisites for the aforementioned deep learning methods, were all resolved by redefining the freeform optimization in the reinforcement-learning framework [54]. This enabled us to propose optical structures with the required responses by utilizing the given learning data to design these structures using deep learning.

The reinforcement-learning method was employed to design distributed Bragg reflectors (DBRs). These reflectors, which consist of alternating layers of materials with high and low refractive indices and are used in optical fiber waveguides and high reflection mirrors, provide controllable reflection spectra. Because it is easy to fabricate DBRs and to control their optical properties, DBRs have received widespread attention for application in optical and optoelectronic devices [55]. In particular, DBRs with transparent conductive oxide (TCO) multi-layers (TiO_2_/SiO_2_) yielded nearly perfect reflector designs at visible and IR frequencies [56]. However, because the reflectance decreases rapidly as the frequency shifts farther from the maximum frequency, the actual stop bandwidth is less than 100 nm. The reflectance is determined by the transfer matrix method (TMM) [57] and by the reflectance index of each layer of material used in the multilayer structure. Reinforcement learning was used to design a DBR with high reflectance, and the size of the multilayer structure was reduced by optimizing the structure.

DBRs designed with the aid of reinforcement learning achieved reflectance of 0.99998 for a size of 500 nm, which was 61.2% smaller than that of the DBRs (1290 nm) designed with the TMM. In addition, the performance of the reflector improved by 20.5% considering that the reflectance of a theoretically designed DBR mirror of the same size (387 nm) is 0.82881.

**Fig 1:**
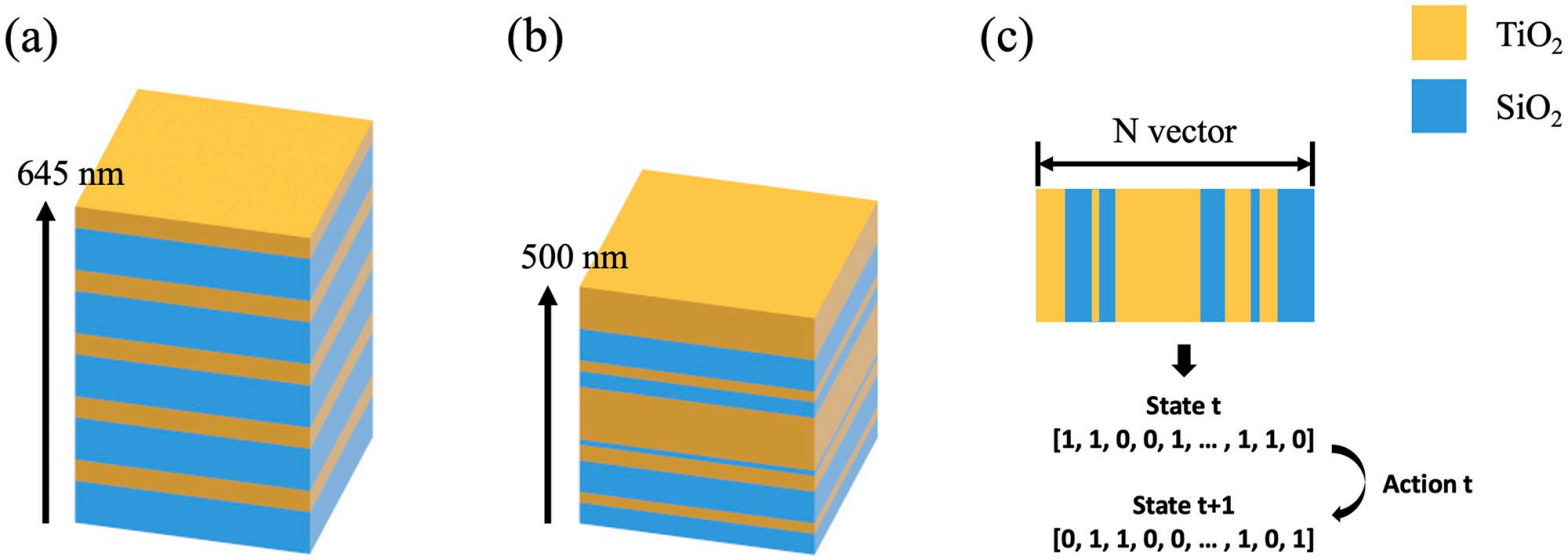
DBR structure consisting of TCO multilayers. The center frequency was designed as 500 nm with the refractive indices of TiO_2_ and SiO_2_ of 2.88 and 1.45, respectively. (a) Design according to TMM. The thicknesses of the TiO_2_ and SiO_2_ layers were 43.4 nm and 85.6 nm, respectively. (b) Structure designed using reinforced learning with an irregular arrangement of TiO_2_ and SiO_2_ layers. (c) Approximation of the structure in (b) as a 1 x N vector composed of zeros and ones for application in reinforcement learning. Here, TiO_2_ corresponds to 1, whereas SiO_2_ is represented by 0. (a) TMM Design, (b) RL Design, (c) State and Action.

Using reinforcement learning, we focused on designing DBRs with a structure different from that of traditional structures. Fig 1(a) shows the design of a DBR based on TMM, with its structure consisting of alternating high- and low-reflectance index materials. The reflectance of such a structure is determined by the reflectance index of each layer, and could be calculated by the TMM as shown in Eq 1 [58].

## Methods

### Problem setup

Using reinforcement learning, we focused on designing DBRs with a structure different from that of traditional structures. Fig 1a shows the design of a DBR based on TMM, with its structure consisting of alternating high- and low-reflectance index materials. The reflectance of such a structure is determined by the reflectance index of each layer, and could be calculated by the TMM as shown in Eq 1 [58].

$$\begin{array}{c} R={\left[\frac{n_{0}{\left(n_{2}\right)}^{2N}-n_{s}{\left(n_{1}\right)}^{2N}}{n_{0}{\left(n_{2}\right)}^{2N}+n_{s}{\left(n_{1}\right)}^{2N}}\right]}^{2} \end{array}$$ (1)

N is the number of repeating pairs of high/low refractive index materials, *n*_0_ the refractive index of air, *n*_1_ and *n*_2_ are the low and high refractive indices of each DBR structure, and *n*_*s*_ the refractive index of the last material. Accordingly, the number of pairs of repeating low/high refractive index materials is one of the factors that determine the overall refractive index. In other words, it is essential to have a certain number of pairs to obtain a refractive index higher than a certain value. As shown in Fig 1b, the structure we designed using reinforcement learning contained irregularly arranged DBRs, which enabled us to reduce the size and improve the performance as the structure was no longer determined by the TMM method.

As shown in Fig 1c, the design of DBRs using reinforcement learning begins by expressing the arrangement of the respective materials by 0 and 1 as a 1 x N vector, where TiO_2_ is expressed as 1 and SiO_2_ as 0. The objective of reinforcement learning is to learn the policy to maximize the total reward from the environment. For this, the design of the DBR was modeled as a series of states and actions an agent can take at each stage. The state refers to the current arrangement of materials (1 x N vector), and each action is intended to change the arrangement of materials. The reward is the reflectivity of the DBR mirror in the current state, which is obtained through simulation, and this reflectivity is calculated using the refractive indices of the materials.

At this moment, the important concepts in reinforcement learning are: state, action, reward, and policy. State refers to the state of the environment in which the agent is located. Action signifies an action the agent can take. Reward represents the response the agent elicits from the environment, which continues to guide the agent’s learning. Lastly, policy means the policy that specifies which action to take in a given state.

### Reinforcement learning

The Deep Q-Network (DQN), a reinforcement-learning algorithm, combines Q-learning with deep learning to enable learning for problems with large state spaces. Q-learning is a method whereby the expected reward, namely the action value (Q-value), is learned when a specific action is taken in a specific state. The Q-value is defined by a pair consisting of a state and action, and allows the policy to maximize the reward by taking high Q-value actions to be learned.

DQN is based on the Q-learning [59] equation, the core learning process of reinforcement learning [58]. This equation expresses the expected reward when a specific action is taken in a specific state. An agent uses the equation to decide which action to take in each state. The Q-learning equation can be expressed by Eq 2:

$$\begin{array}{c} Q\left(s,a\right)=r+\gamma {\text{max}}_{a^{,}}Q\left(s^{\prime },a^{\prime }\right) \end{array}$$ (2)

where *Q*(*s*, *a*) indicates the optimal action value (Q-value) when an action a is taken in the state *s*, *r* is the immediate reward for the present state and action, *γ* is the discount coefficient for the future reward, *s*′ is the next state, *a*′ is the next action, and ${\text{max}}_{a^{,}}Q\left(s^{\prime },a^{\prime }\right)$ the maximum Q-value expected among all the actions possible in the next state.

The calculated Q-values are learned through the neural network and the learning process is designed to minimize the loss function, where the loss function indicates the difference between the learned Q-value and the target Q-value calculated through the Q-learning equation. The loss function can be expressed by Eq 3 [60]:

$$\begin{array}{c} \text{Loss}={\left(Q\left(s,a;\theta \right)-(r+\gamma {\text{max}}_{a^{,}}Q\left(s^{\prime },a^{\prime };\theta^{\prime }\right))\right)}^{2} \end{array}$$ (3)

where *θ* indicates the neural network parameter, *θ*′ the target network parameter. The parameters of the neural network are updated iteratively while the loss function is minimized using the gradient descent method.

### Double Dueling Deep Q-Network(D3QN)

In this study, reinforcement learning was carried out by using the Double Dueling Deep Q-Network (D3QN). D3QN is a combination of two methods: the Dueling Architecture, a reinforcement-learning method, and Double Q-Network (DQN). This combination makes it possible to utilize all the advantages of each of these two structures. The dueling architecture separates the state-value function from the action-value function, and allows each state value and the relative value of each action to be estimated independently and accurately. This enables an agent to more accurately judge which action is the most effective.

DQN solves the problem of overestimation of the Q-value by lowering the tendency of a reinforcement-learning agent to overestimate the value of a specific action. Solving this problem is important, because it induces an agent to preferably choose a specific action, which is undesirable.

**Fig 2:**
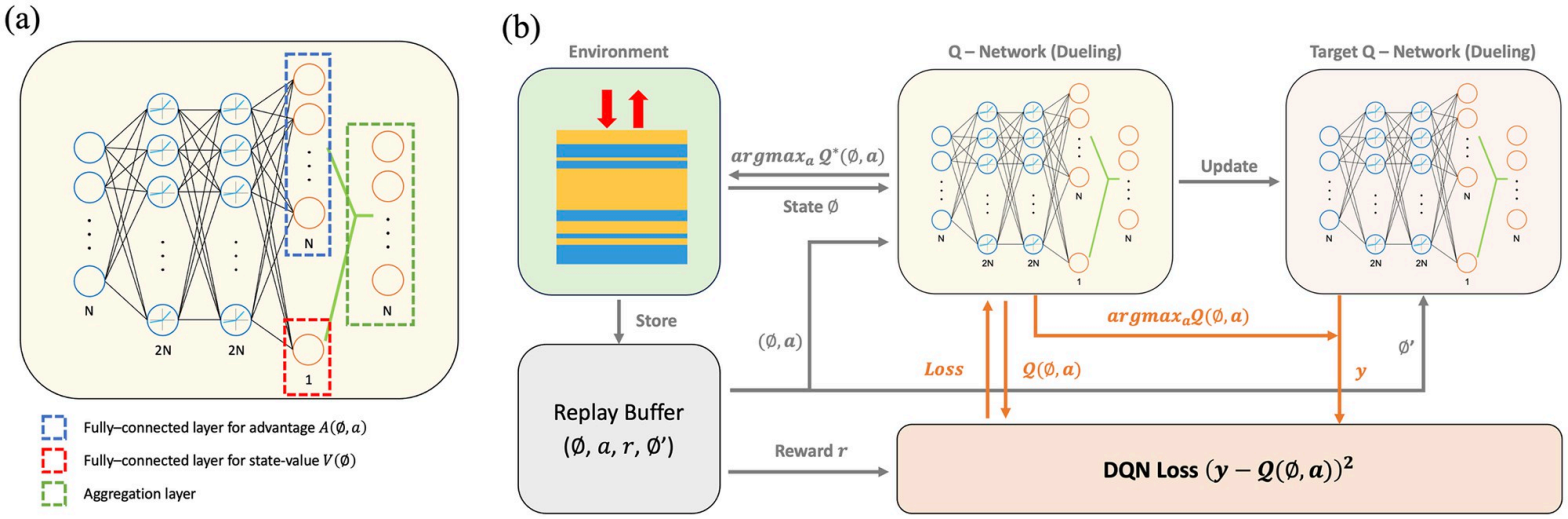
Schematic diagrams of dueling architecture and double architecture. (a) The dueling architecture separates and processes the state value function *V*(*s*) and the action value function *A*(*s*, *a*), thereby enabling an agent to separately learn the relative values when a specific action is taken in each state and at each value. The final Q-value is calculated by adding the two functions. (b) DQN mechanism. Overestimation of the Q-value is prevented because the two independent Q-networks separate the selection of the action and estimation of the Q-value. This method prevents the reinforcement-learning agent from taking overly optimistic actions and stabilizes the learning process. (a) Dueling architecture, (b) DQN mechanism.

Dueling Architecture divides the Q-value an agent is learning into two parts, as illustrated in Fig 2a. The first is a state-value function *V*(*s*), and the other is an action-value function *A*(*s*, *a*). The former function represents the value of a specific state, whereas the latter function represents the relative value when a specific action is taken in a specific state. The two values are added to obtain the final Q-value, and can be expressed by Eq 4 [61].

$$\begin{array}{c} Q\left(s,a;\theta ,\theta^{\prime },\xi \right)=V\left(s;\theta ,\xi \right)+(A\left(s,a;\theta ,\theta^{\prime },\xi \right)-\text{avg}(A(s,a^{\prime };\theta ,\theta^{\prime },\xi ))) \end{array}$$ (4)

where *θ* indicates the neural network parameter, *θ*′ the target network parameter, and E the expected values. The parameters of the neural network are updated iteratively while the loss function is minimized using the gradient descent method. where *s*, *a* represents each pair consisting of a state and action, *θ* and *θ*′ the parameters of the state-value function and the action-value function, respectively, and *ξ* is the shared parameters.

DQN uses two Q-networks to prevent it from overes-timating the Q-value [61], which could induce an agent to take an overly optimistic action, and destabilize the process of learning. As shown in Fig 2b, the first is used to select an action (basic Q-network), and the other to estimate the Q-value of the action taken (target Q-network). An action is selected based on the basic Q-Network, as expressed by Eq 5.

$$\begin{array}{c} a={\text{argmax}}_{a}Q\left(s^{\prime },a;\theta \right) \end{array}$$ (5)

where *θ* is the parameter of the basic Q-Network. Subsequently, the Q-value of the action taken is estimated by using the target Q-Network [62]:

$$\begin{array}{c} Q\left(s,a\right)=r+\gamma Q(s^{\prime },{\text{argmax}}_{a}Q\left(s^{\prime },a;\theta \right);\theta^{\prime }) \end{array}$$ (6)

where *θ*′ represents the parameter of the Q-Network, *r* is the reward, *γ* is the discount coefficient, and *s*′ is the next state.

The combination of various reinforcement-learning methods is known to be a successful way to solve problems using reinforcement learning because the advantages of each of the respective method are exploited [63]. As such, the most effective action is taken in each state by combining the two kinds of structures mentioned before. In this regard, D3QN is able to evaluate the value of the action taken more accurately to effectively solve the problem presented by reinforcement learning.

The pseudocode of D3QN is provided in Algorithm 1. Initially, the algorithm initializes the Q-network and Q-target network to arbitrary weighted values. Q-Network has two streams that separate and process the state-value function and action-value function, respectively. Additionally, the replay buffer to be used in the learning process is initialized.

**Algorithm 1** Double Dueling Deep Q-Network

1: Initialize Q-network and Q-target-network with random weights; Q-network will have dual streams for value and advantage functions.

2: Initialize memory replay buffer D.

3: **for** each episode = 1, …, *N* **do**

4:  Initialize the environment

5:  **for** each step = 1, …, *M* in episode **do**

6:   Get current state *ϕ*_*t*_

7:   With probability epsilon *ϵ* select a random action *a*_*t*_,

8:   else $a_{t}={\text{argmax}}_{a}Q^{*}\left(\phi_{t},a\right)$ using current Q-network

9:   Execute action *a*_*t*_ in the environment and observe reward *r*_*t*_ and next state

10:   *ϕ*_*t*+1_

11:   Store transition (*ϕ*_*t*_, *a*_*t*_, *r*_*t*_, *ϕ*_*t*+1_) in D

12:   **if** D size > minimum required size for training **then**

13:    Sample a batch (*ϕ*_*j*_, *a*_*j*_, *r*_*j*_, *ϕ*_*j*+1_) from D and calculate target *y*_*j*_

14:    Determine $a^{\prime }={\text{argmax}}_{a}Q\left(\phi_{j+1},a^{\prime }\right)$ using current Q-network

15:    Set $y_{j}=\{\begin{array}{ll} r_{j} & \text{for}\;\text{episode}\;\text{is}\;\text{done}.\\ r_{j}+\gamma {\text{argmax}}_{a^{\prime }}Q_{\text{target}}(\phi_{j+1},a^{\prime }) & \text{for}\;\text{episode}\;\text{is}\;\text{not}\;\text{done} \end{array}$

16:    Update Q-network by minimizing (*y*_*j*_ − *Q*(*ϕ*_*j*_, *a*_*j*_))^2^

17:    where *Q*(*ϕ*, *a*) = *V*(*ϕ*) + (*A*(*ϕ*, *a*) − avg(*A*(*ϕ*, *a*)))

18:   **end if**

19:   **if** *M*% target network update frequency == 0 **then**

20:    Update Q-target-network by copying weights from Q-network

21:   **end if**

22:   *ϕ*_*t*_ = *ϕ*_*t*+1_

23:  **end for**

24: **end for**

Whenever each episode starts, the environment is initialized, and a series of steps are processed in each episode. In each step, the present state is acquired, and an arbitrary action is selected, or the optimal action is selected using the present Q-network according to the *ϵ*-Greedy policy. In accordance with this policy, which is used in reinforcement learning, a random action is selected according to a constant probability *ϵ*, whereupon the action is taken; otherwise, the action with the highest value based on the current information is taken. The selected action is processed in the environment, and, as a result, the reward and the next state are observed. These transitions (present state, action, reward, and next state) are stored in the replay buffer.

When the size of the replay buffer reaches the mini-mum size required for learning, batches are sampled from the buffer, and the target value for each sampling is calculated. At this point, the optimal action is decided at the next state using the current Q-network, after which the Q-value of this action is estimated using the target Q-network. The Q-network is updated using the calculated target value, whereby the method to estimate the action value of an agent is improved.

Lastly, the present weighted value of the present Q-network is periodically assigned to the target Q-network to update its weighted value, which plays an important role in solving the problem of overestimation of the Q-value in double Q-learning. This process is carried out iteratively in each episode and step, which enables the agent to learn gradually through this interaction with the environment.

This approach enables D3QN to solve the problem using reinforcement learning, which contributes to improving the performance by utilizing all the advantages of both Dueling Architecture and Double Q-Network. Because the target Q-network is updated at a low frequency, this network limits the instability that could arise during learning. This process stabilizes the learning and mitigates the problem of overestimating the Q-value. Moreover, the *ϵ*-value of the *ϵ*-Greedy policy could decrease as learning proceeds. Although various actions could initially be taken because of the preference for exploration, the agent would take the optimal action based on the learned knowledge as learning progresses.

D3QN attempts to minimize the shortcomings and maximize the advantages of each method by combining Dueling Architecture and Double Q-Network. The former network enhances the accuracy of the action values by independently estimating the state value and action value. On the other hand, DQN mitigates the problem of overes-timation and stabilizes the learning process.

In conclusion, D3QN is an algorithm that can be used effectively in various problem situations of reinforcement learning. D3QN can learn the optimal policy fast and stably. This suggests that D3QN is suitable for use in various real applications.

## Results

### Experimental details

D3QN was trained by Adam optimizer of 1e-3 learning rate, 512 batch size, and without weight decay for 12 hours. We employ an epsilon-greedy policy that decreases linearly over 2 million steps to a minimum of 0.001 for action selection. The network, comprised of two fully connected layers with Leaky ReLu, initialized orthogonally to improve convergence stability. We set the discount coefficient *γ* to 0.99.

### Performance of D3QN

**Fig 3:**
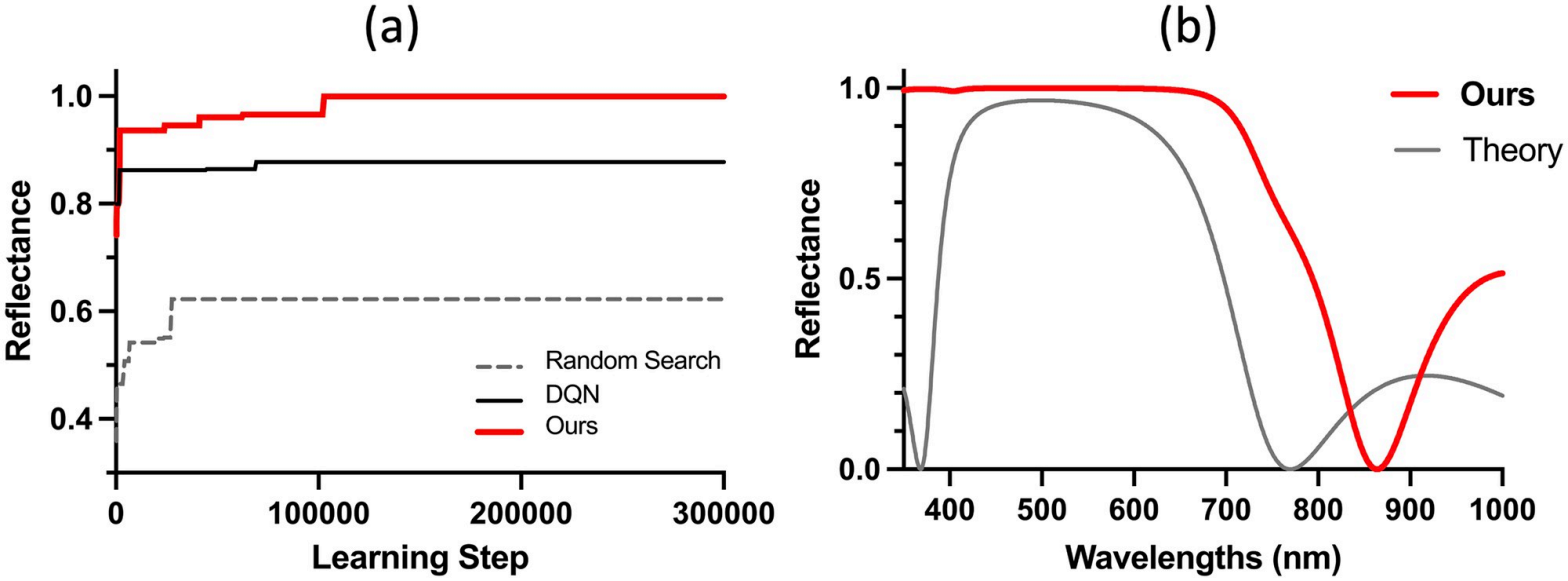
Experimental and designed reflectance of DBR mirror sized 516 nm (500 nm Bragg wavelength). (a) Effect of the number of learning steps on the reflectance. The reflectance of D3QN (blue line) converges faster than those of DQN and Random Search. The maximum reflectance of D3QN was also the highest. (b) Designed reflectance of four pairs (516 nm) based on the TMM. The reflectance designed with D3QN (blue curve) is superior to that obtained with TMM (black curve). (a) D3QN vs DQN, (b) D3QN vs TMM (516nm).

The D3QN algorithm outperforms original DQN and TMM. Fig 3 shows the reflectance of the DBR mirror that was designed using D3QN. The wavelength at the peak reflectance (Bragg wavelength) was 500 nm, and the DBR mirror size was designed to be 516 nm. This size corresponds to four pairs according to the TMM. The learning process is plotted in Fig 3a. Three methods (D3QN (this study), DQN, and Random Search) were compared, and the maximum reflectance values were compared as a function of the number of learning steps of each method. The maximum reflectance values decreased in the order of D3QN (0.9997), DQN (0.8778), and Random Search (0.6231). The convergence speed of D3QN was also the highest. Particularly, the maximum reflectance of D3QN was higher by 0.1219 compared to that of DQN, and by 0.3766 compared to that of Random Search. Fig 3b shows the reflectance as a function of the wavelength. The maximum reflectance of D3QN was higher by 0.0322 than that of TMM, and the bandwidth was twice as wide.

### Transfer learning with different size and Bragg-wave length

**Fig 4:**
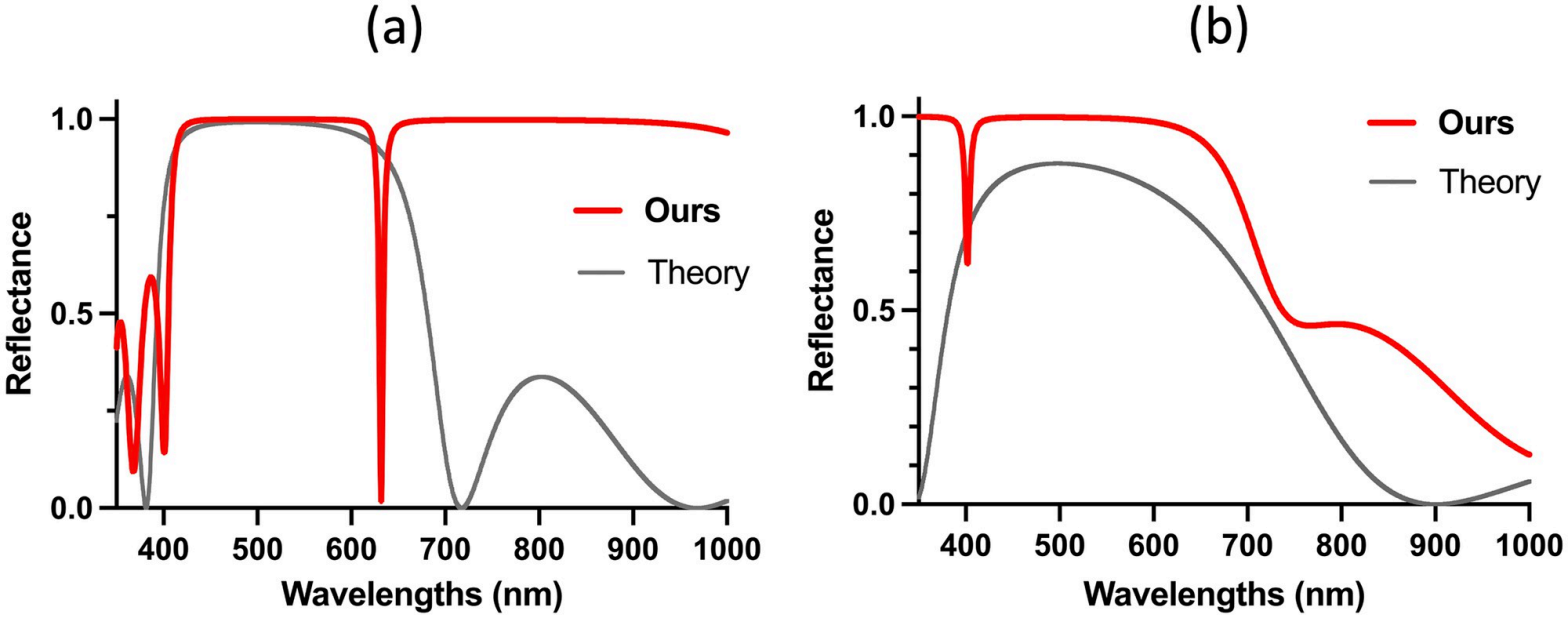
Designed reflectance of DBR mirrors with sizes of 645 and 387 nm (500 nm Bragg wavelength). (a) Designed reflectance of 645 nm size corresponding to five pairs based on the TMM (black curve). The designed reflec-tance of D3QN (blue curve) outperforms that of the TMM. (b) Designed reflectance of three pairs (387 nm). The designed reflectance of D3QN (blue curve) is superior to that of the TMM. (a) D3QN vs TMM (645nm), (b) D3QN vs TMM (387nm).

**Fig 5:**
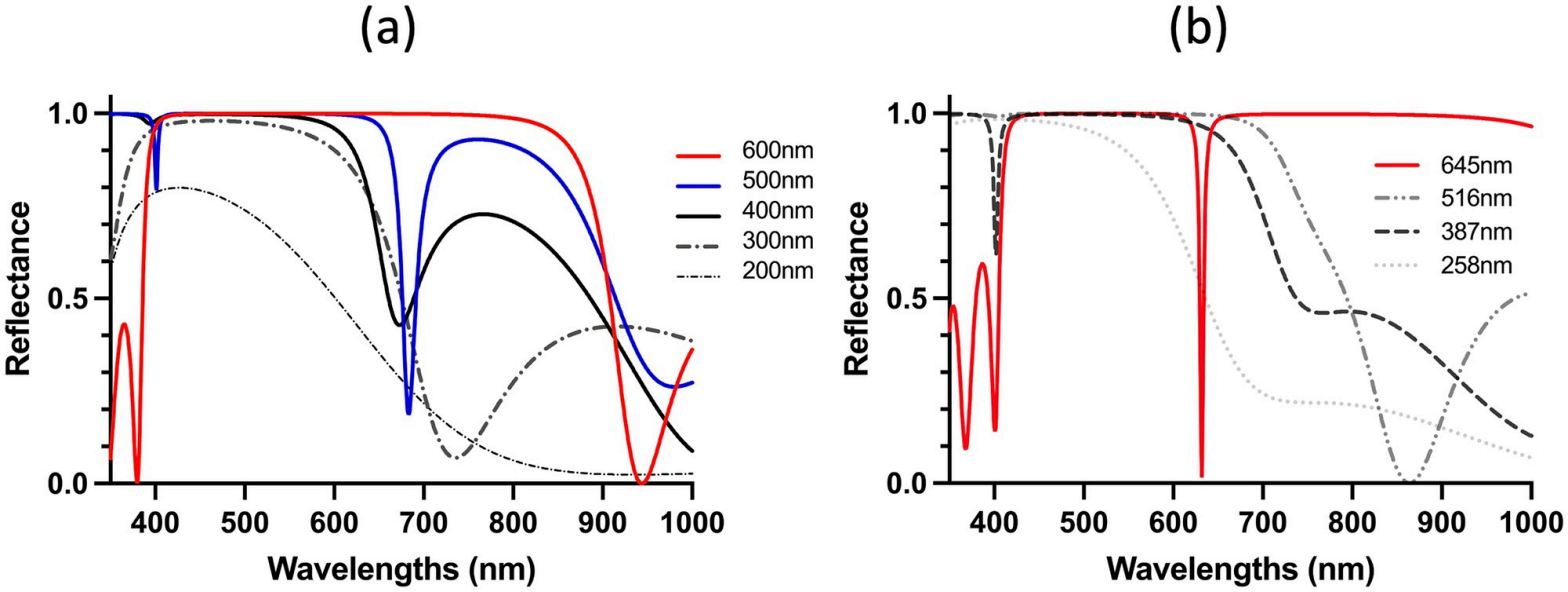
Designed reflectance for 500 nm Bragg wavelength. (a) Effect of DBR mirror size (200—600 nm) on the reflectance. The 600 nm size mirror (red curve) has the best reflectance. (b) Effect of the number of pairs on the reflectance (two pairs (258 nm) to five pairs (645 nm). The reflectance of five pairs is the best.

We adopt transfer learning [64] to improve the sample efficiency of D3QN. In transfer learning, a neural network pre-trained for a specific Bragg wavelength and DBR size can be utilized to design DBR mirrors with different Bragg wavelengths and DBR sizes. There are two methods of transfer learning to enhance the D3QN algorithm. The first method involves adjusting the target DBR size while keeping the Bragg wavelength constant (Figs 4 and 5). In contrast, the second method alters the target Bragg wavelength while keeping the DBR size consistent (Fig 1). These strategies enable effective exploration of parameter spaces, thereby improving the adaptability of the D3QN model to different optical conditions.

Fig 4 shows the reflectance of DBR mirrors with sizes of 645 nm and 387 nm. These sizes correspond to five pairs and three pairs based on the TMM, respectively. For these conditions, D3QN was trained using a pre-trained model for 516nm DBR size and a 500nm Bragg wavelength, as illustrated in Fig 3. Both of these results show that the D3QN designs exhibited higher reflectance and wider reflection bandwidth than the designs by the TMM. Fig 5 shows the reflectance of the designs of DBRs with various sizes (600 200 nm). The reflectance of most of the sizes was higher than 0.9 and thus superior to those designed by the TMM.

### Generalization and comparative performance analysis of the D3QN method

To generalize the scope of the D3QN method, the effects of various Bragg wavelengths and various mirror sizes were investigated, and the results are presented in Table 1. In most of the cases, the reflectance was higher than 0.9. Notably, the reflectance was higher than 0.9999 when the size of the DBR mirror exceeded 92.1% of the Bragg wavelength, and the reflectance was 1 when the size was larger than five pairs based on the TMM.

**Table 1:** Effects of mirror size (200—700 nm) and Bragg wavelength (400—900 nm) on the reflectance.

Reflectance										
Bragg-wage length	Size (nm)									
	700	645	600	516	500	400	387	300	258	200
400nm	1	1	1	0.99996	0.99997	0.99997	0.99997	0.99819	0.99276	0.96333
500nm	1	0.99996	0.99998	0.99998	0.99997	0.99220	0.99873	0.98503	0.97128	0.82846
600nm	0.99998	0.99998	0.99997	0.99982	0.99968	0.99476	0.99276	0.96333	0.85583	0.72001
700nm	0.99997	0.99994	0.99981	0.99785	0.99717	0.98328	0.98093	0.85354	0.77661	0.72001
800nm	0.99987	0.99936	0.99819	0.99276	0.98958	0.96333	0.95082	0.79009	0.69460	0.36734
900nm	0.99879	0.99726	0.99478	0.98346	0.98135	0.89378	0.85583	0.72001	0.55336	0.21059

The reflectance corresponding to each Bragg wavelength is listed against the size of the DBR mirror in the leftmost column.

Table 2 provides a comparative analysis of the reflectance performance of the D3QN method against other optimization techniques, including Random Search, Greedy Optimization, Double DQN, and Dueling DQN. This comparison was conducted across various DBR sizes (200 nm, 300 nm, and 400 nm) and Bragg wavelengths (400 nm, 600 nm, and 800 nm).

**Table 2:** Comparative reflectance performance across different mirror sizes and Bragg wavelengths. * the best results are highlighted.

Reflectance						
DBR Size	Bragg wavelength	Methods				
		D3QN(ours)	Random Search	Optimization	Double DQN	Deuling DQN
400nm	400nm	**0.99997**	0.64315	0.76915	0.89624	0.87346
	600nm	**0.99476**	0.47380	0.79318	0.90317	0.85371
	800nm	**0.98958**	0.33281	0.81357	0.94315	0.92371
300nm	400nm	**0.99997**	0.71354	0.88341	0.86317	0.90371
	600nm	**0.99276**	0.71352	0.91355	0.92381	0.93517
	800nm	**0.96333**	0.61379	0.76513	0.81376	0.79351
200nm	400nm	**0.96333**	0.66355	0.88614	0.91333	0.91333
	600nm	**0.72001**	0.46814	0.49381	0.63519	0.58731
	800nm	**0.36734**	0.12044	0.21337	0.33994	0.31077

This table presents the reflectance performance of the D3QN method in comparison to other optimization methods, including Random Search, Greedy Optimization, Double DQN, and Dueling DQN. The results are shown for various DBR sizes (200 nm, 300 nm, and 400 nm) and Bragg wavelengths (400 nm, 600 nm, and 800 nm). The best reflectance results for each configuration are highlighted, demonstrating the superior performance of the D3QN method in most cases.

The empirical results demonstrate that the D3QN method consistently outperforms the other methods, achieving superior reflectance values across a majority of configurations. For instance, at a DBR size of 400 nm and a Bragg wavelength of 400 nm, the D3QN method attained a reflectance of 0.99997, markedly higher than the reflectance achieved by Random Search (0.64315) and Greedy Optimization (0.76915). Similarly, for a DBR size of 300 nm and a Bragg wavelength of 600 nm, the D3QN method achieved a reflectance of 0.99276, outperforming Double DQN (0.92381) and Dueling DQN (0.93517).

These findings underscore the efficacy of the D3QN method in optimizing DBR designs across diverse sizes and wavelengths. The robustness and adaptability of the D3QN approach are further highlighted by its ability to maintain high reflectance levels, often exceeding 0.9, under varied conditions.

## Conclusion

This study led to the proposal of a new method, D3QN, which was developed to replace the traditional TMM method for DBR mirror design. D3QN was demonstrated to produce design results superior to those of TMM. D3QN enables the structure of a DBR mirror to be learned using reinforcement learning, and allows various parameters such as the material and shape to be changed according to the user’s intentions. This paper primarily focuses on 1D DBR structures. While extending the approach to 2D and 3D structures is mentioned, it is not explored in this study. In future, the proposed method could be expanded to include 2D and 3D design structures. Although the design would become more complex in this case, this could be solved by D3QN or similar solutions. Additionally, as the current work is simulation-based, future efforts should include fabricating and experimentally testing the D3QN-optimized DBR designs to validate their practical applicability and address potential manufacturing challenges, including exploring the tolerance of these designs to manufacturing errors.
